# Supplementary material for: Transcranial Magnetic Stimulation as a Tool to Promote Smoking Cessation and Decrease Drug and Alcohol Use
Source: Brain Sci. 2023 Jul 14;13(7):1072. doi: 10.3390/brainsci13071072 (PMC10377606; doi:10.3390/brainsci13071072)
Supplement: Supplementary file 1 [file brainsci-13-01072-s001.zip › brainsci-2416399-supplementary.pdf]

Transcranial Magnetic Stimulation as a tool to promote smoking cessation and decrease drug and alcohol use.

Tal Harmelech, Colleen A. Hanlon, and Aron Tendler

Supplementary Tables:

Table S1. Summary table of TMS studies in smoking addiction. page 2

Table S2. Summary table of TMS studies in alcohol use disorder. page 7

Table S3. Summary table of TMS studies in cocaine use disorder. page 12

Table S1. Summary table of TMS studies in smoking addiction.

Study	Design	Sample	Protocol / Stimulation Parameters	Assessments	Results
**Figure-8 coil**					
Johann et al. 2003	Randomized, double-blind, crossover assessed whether HF rTMS to left DLPFC could modulate subjective tobacco craving	N=11 (23–55 yo) tobacco-dependent treatment-seeking smokers	1 active/sham session. 20 Hz over left DLPFC, 90% rMT, 20 trains, 2.5 sec on, 42.5 sec off, 1,000 pulses/sess., 14 min. Participants required to abstain from smoking 12 hrs before rTMS session	VAS and FTND	Active rTMS significantly decreased craving level vs. sham 30 min following rTMS
Eichhammer et al. 2003	Randomized, double-blind, crossover active, or sham HF rTMS to left DLPFC aimed at replicating results of Johann et al.	N=14 treatment-seeking tobacco-dependent smokers	2 active 20 Hz rTMS over left DLPFC and 2 sham sessions across 4 days, 90% rMT, 20 trains, 2.5 sec on, 42.5 s off., 1,000 pulses/sess., 14 min. Participants required to abstain from smoking 12 hrs before rTMS session and freely smoked in 6 hr time period	VAS. Number of cigarettes	Craving levels did not change. Reduced cigarette smoking significantly in active vs. sham.
Amiaz et al. 2009	Randomized, double-blind, sham-controlled study evaluated effects of HF rTMS over left DLPFC combined with smoking/neutral cues on cigarette consumption, dependence, and craving	N=48 chronic smokers with nicotine dependence (20– 70 yo, at least 20 cigarettes/day) motivated to quit smoking	10 daily sessions of 10 Hz rTMS over left DLPFC, 100% rMT, 20 trains, 5 sec on, 15 sec off. Followed by maintenance phase for additional month (3 rTMS sessions (active/sham) in 1st week on alternate days followed by 1 session per week during following 3 wks.	VAS with sTCQ<br>Urine cotinine levels on screening day and on days 1, 5, and 10, then at each maintenance session.<br>mFTND conducted 6 months after treatment termination	10 daily active DLPFC rTMS sessions, independent of exposure to smoking pictures, reduced cigarette consumption, and craving vs. sham.
Rose et al. 2011	Controlled within-subject study rTMS over SFG investigated effects of rTMS on subjective responses to smoking vs. neutral cues and to controlled presentations of cigarette smoke	N=15 healthy smokers (18–50 yo; at least 15 cigarettes/day)	Participants exposed to 1 of 3 conditions: 3 periods of 2 min and 30 sec, 10 Hz rTMS to SFG (FPz); 1 Hz rTMS to SFG; and 10 Hz rTMS to motor cortex at 90% rMT concurrently with presentation of smoking, control cues, and smoking cigarettes. Smokers required to smoke at least 10 cigarettes per day and have no plans to quit smoking in next 30 days.	. Craving ratings assessed by Shiffman–Jarvik questionnaire	10 Hz SFG rTMS resulted in increased cue-induced craving but lower craving during presentation of neutral cues. Craving ratings after smoking cue presentations elevated in 10 Hz SFG condition vs. 1 Hz SFG rTMS or motor cortex whereas craving after neutral cue presentations reduced.
Wing et al. 2012	10-week, double-blind, randomized, sham-	N=15 heavily dependent	20 sessions. 5 sess./week (in weeks 1–4) bilateral 20 Hz	Smoking (self-report and breath carbon monoxide	Despite attenuation of tobacco cravings,

	controlled active or sham bilateral HF rTMS assessed efficacy on smoking cessation	treatment-seeking smokers (18 – 60 yo; 10 cigarettes/day) with schizophrenia or schizoaffective disorder.	rTMS over DLPFC, 90% rMT, 50 trains, 1.5 sec on, 30 sec off, 750 pulses on each hemisphere as adjunct to weekly group therapy and TN: 21 mg provided in weeks 3–9	[CO] levels), PANSS, TQSU, MNWS	rTMS did not increase abstinence rates.
Sheffer et al. 2013	Single-blind, within-subjects study	N=47 smokers (19–55 yo) with no intention to quit and N=19 non-smokers	3 sessions. each of 20 Hz rTMS over left DLPFC, 110% MT, 1 sec on, 20 sec off; 900 pulses/sess., 10 Hz, 20 Hz, 110% rMT, 1 sec on, 20 sec off; or sham rTMS separated by 48 h. Participants were to abstain from smoking 6 h before experiment.	Delayed discounting FTND	20 Hz rTMS over left DLPFC decreased discounting monetary gains but increased discounting monetary losses. Stimulation had no effect on cigarette consumption.
Li et al. 2013	Randomized, double-blind, sham-controlled, crossover assessed whether HF left DLPFC rTMS vs. sham would temporarily reduce subjective craving triggered by exposure to smoking cues	N=16 (21–60 yo) non-treatment seeking, nicotine-dependent participants (21–60 yrs; 10 cigarettes/d)	1 sess., 15 min, 10 Hz rTMS over left DLPFC rTMS, 100% rMT, 5 sec on, 10 sec off, 3,000 pulses/sess. Participants required to abstain from smoking for 2rs prior to experiment.	FTND, QSU-B, MWSR	Active left DLFPC rTMS but not sham significantly reduced subjective craving induced by smoking cues in nicotine-dependent participants from baseline
Pripfl et al. 2014	Sham-controlled study investigated EEG delta power changes induced by HF left DLPFC rTMS and its relation to cue-induced nicotine craving	N=14 healthy smokers with nicotine addiction and nicotine-deprived smokers	1 active session of 10 Hz rTMS for active over left DLPFC and 1 sham session	Craving and resting state EEG delta power	Both craving and EEG delta power significantly lower after active rTMS vs. sham across entire post-stimulation time period. HF rTMS over left DLPFC reduced nicotine craving in short-term abstinent smokers
Prikryl et al. 2014	Double-blind, randomized placebo-controlled study 10 Hz rTMS over left DLPFC assessed effects on cigarette consumption	N=35 schizophrenia smokers not seeking treatment on stable anti-psychotic medication (18– 60 yo, at least 10	21 sessions. 10 Hz rTMS over left DLPFC, 110% rMT, 20 trains, 10 sec on, 30 sec off, 2,000 pulses/sess.	Cigarette consumption, PANSS, MADRS, CDSS	Cigarette consumption significantly lower in active vs. sham as early as 1st treatment week.

		cigarettes/day for last 2 yrs			
Trojak et al. 2015	Three-phase prospective, placebo-controlled, randomized study of combined rTMS with NRT	N=37 (18–65 yo) with desire to quit smoking	During phase 1 only, 10 sess. (2 weeks) 1 Hz rTMS over right DLPFC MRI-neuro-navigated target, 120% rMT, 6 trains, 60 sec on, 30 sec off, 360 pulses/sess. Phase 2 was a 4-week period of NRT alone (14 mg/24-h for 2 weeks then 7 mg/24-h patches for 2 weeks). Phase 3 was 6 wk follow-up phase without any treatment	Cigarette consumption. Craving with VAS, QSU. Continuous abstinence based on self-report and verified by CO<10 ppm and assessed at weeks 2, 6, and 12. PANSS and adverse events assessed weekly	Active rTMS but not sham significantly decreased self-reported compulsive behaviors related to craving. Active rTMS but not sham significantly decreased self-reported compulsive behaviors related to craving. Active rTMS resulted in significantly reduced level of craving and more abstinent subjects vs. sham.
Sheffer et al. 2018	Double-blind, sham-controlled, randomized study of combined rTMS and evidence-based self-help intervention	N=29 (21-65 yo) who smoked 5-20 cigarettes/day, abstinent for 24h, motivated to quit, and not using cessation medications	8 sessions across 2 weeks of 20Hz rTMS over the left DLPFC or sham, 110% rMT, 45 trains, 1 sec on, 20 sec off, 900 pulses/sess, 16 min. Stimulation site was located using the 6cm rule and neuro-navigation. Participants were required to achieve biochemically confirmed 24-hour abstinence prior to the first stimulation session. Participants read the 8 self-help booklets, in order, during the stimulation sessions and were encouraged to continue to read the material in the lab and/or at home.	Daily cigarette use was assessed weekly by telephone. FTND, MNWS, ARME, and Delay discounting Abstinence from smoking were assessed with CO.	Active rTMS vs. sham significantly decreased delay discounting of $100 and $1000, significantly reduced the relative risk of relapse 3-fold, significantly increased abstinence rates (active 50% vs. sham 15.4%, p=.05), and increased uptake of the self-help intervention.
Chang et al. 2018	Open-label, 10 days rTMS treatment and 25 days follow-up	N=14 (29-57 yo) treatment seeking who smoked for more than 5 years (and more than 10 cigarettes/day)	The rTMS treatment started after 24-h abstinence from smoking. 20 Hz rTMS sequentially over the left DLPFC and the SMFC. 90% rMT, 1000 pulses/session	CO levels, withdrawal (MNWS), craving scales, and neuroimaging data were collected	A significant smoking craving reduction and resting brain activity reduction measured by the cerebral blood flow (CBF) and brain entropy (BEN) were observed after 10 days of 20 Hz rTMS treatments compared to baseline.
Li et al. 2020	Double-blind, sham-controlled, 10 daily	N=42 (18-60 yo) treatment-	10 daily sessions across 2 weeks of either active or	Primary outcome was reduction in biochemically	Active rTMS significantly reduced

	MRI-guided rTMS sessions across two weeks over the left DLPFC paired with craving cues to reduce cigarette consumption and induce smoking cessation	seeking nicotine-dependent smokers (≥10 cigarettes/day)	sham MRI-guided rTMS (10Hz,100% rMT, 5 sec on, 10 sec off, 3000 pulses/session) over the left DLPFC concurrently with video smoking cues, 15 min. Participants were instructed to abstain from smoking at least 2 h before each treatment.	confirmed (urine cotinine, CO) cigarette consumption. VAS, FTND, QSU-B, MNWS, and urine cotinine levels follow-up). Carbon monoxide (CO) levels were measured before each TMS treatment. Participants asked to select a TQD A successful quit was defined as at least 2 days of abstinence and CO < 5 parts per million (ppm). Continuous abstinence was assessed at 7-day and 4-week visits after the last rTMS session.	the number of cigarettes/day during the treatment and at 1-month follow-up; increased the likelihood of quitting by the target quit date (23.81% vs. 0%); reduced mean craving throughout treatments and at follow-up.
**Deep TMS H4 Coil**					
Dinur-Klein et al. 2014	Double-blind, sham-controlled, 13 daily sessions of high/low frequency or sham Deep TMS over lateral prefrontal and insular cortices, with/without smoking cues	N=115 heavy smokers (≥20 cigarettes/day) who failed previous antismoking treatments, and self-reported symptoms of mild chronic obstructive pulmonary disease	13 daily sessions of high frequency (33 trains of 10 Hz each lasting 3 seconds with an intertrain interval of 20 seconds. Total treatment duration was 760 seconds with 990 pulses), low frequency (600 continuous pulses at 1 Hz), or sham Deep TMS, over lateral prefrontal and insular cortices, with or without presentation of smoking cues.	Cigarette consumption was evaluated by measuring cotinine levels in urine samples and recording participants' self-report. Craving.	High (but not low) frequency Deep TMS treatment significantly reduced cigarette consumption and nicotine dependence. The combination of this treatment with exposure to smoking cues enhanced reduction in cigarette consumption leading to an abstinence rate of 44% at the end of treatment and an estimated 33% 6 months following the treatment.
Zangen et al. 2021	Double-blind, sham-controlled, 15 daily Deep TMS sessions followed by 3 weekly sessions over the lateral prefrontal and insular cortices paired with cue-induced craving procedure. Up to 12 weeks follow-up.	N=262 chronic smokers meeting DSM-5 criteria for tobacco use disorder, who have failed at least one prior attempt to quit (68% failed at least 3 prior attempts)	3 weeks of daily bilateral active or sham Deep TMS to the lateral prefrontal and insular cortices, followed by once weekly Deep TMS for three weeks. Each Deep TMS session was administered following a cue-induced craving procedure, and participants were monitored for a total of six weeks. Those in abstinence were monitored for an additional 12 weeks. Each Deep TMS session consisted of 60 trains of 30 pulses (total of 1,800 pulses)	The primary endpoint was a four-week continuous quit rate (CQR) until week 18 in the intent-to-treat (ITT) efficacy set. It was assessed through participants' self-reports and verified with urine cotinine levels.	In the ITT analysis set (N=234) the CQR until week 18 was 19.4% following active and 8.7% following sham Deep TMS ($\chi2$=5.655, p=0.017). Among completers (N=169), the CQR until week 18 was 28% and 11.7%, respectively ($\chi2$=7.219, p=0.007). The reduction in cigarette consumption and

			applied at 10 Hz (3 sec each train) with 15 s inter-train intervals, at 120% rMT		craving was significantly greater in the active than in the sham group as early as two weeks into treatment.
Moeller et al. 2022	Double-blind randomized sham-controlled study with multimodal imaging	N=20 schizophrenia patients with comorbid TUD	3 weeks (17 sessions) of Deep TMS or sham HF (10 Hz) stimulation to the lateral prefrontal and insular cortices. Each session included 60 trains, each lasting 3 sec and interleaved with a 15 s delay, delivered over 20 min, at 120% rMT	To test for the therapeutic potential (smoking addiction disruption) of Deep TMS, participants completed the laboratory-based McKee Smoking Lapse Test at baseline and after the last Deep TMS session. Arterial spin labeling was used to measure insula cerebral blood flow as an index for target engagement and resting state functional connectivity to measure modulation of insula-centric networks. Both were acquired before the self-administration at baseline and after the last Deep TMS session, as well as after the first Deep TMS session.	Participants assigned to active Deep TMS were slower to initiate smoking their first cigarette following treatment compared with sham (p=0.03), consistent with smoking disruption. The imaging analyses did not reveal significant Time × Group interactions, but effects were in the anticipated directions (i.e., more pronounced following active vs. sham stimulation). In arterial spin labeling analyses testing for target engagement, an overall decrease in insula blood flow, measured during a post-treatment MRI versus baseline, was numerically more pronounced in the active Deep TMS group than sham. In fMRI analyses, resting-state connectivity between the insula and default mode network showed a numerically greater change from baseline in the active Deep TMS group than sham, consistent with a functional change to insula circuits.

Table S2. Summary table of TMS studies in alcohol use disorder.

Study	Design	Sample	Protocol / Stimulation Parameters	Assessments	Results
**Figure-8 coil**					
Mishra et al. 2010	Prospective, single-blind, sham-controlled study	N=45 patients with alcohol dependence syndrome (according to ICD-10 DCR), with Clinical Institute of Withdrawal Assessment in Alcohol Withdrawal (CIWA-Ar) scores ≤10	Patients were allocated to active and sham rTMS in a 2:1 ratio, such that 30 patients received active and 15 patients sham rTMS to the right DLPFC (10 Hz frequency, 4.9 seconds per train, intertrain interval of 30 seconds, 20 trains per session, total 10 sessions)	The Alcohol Craving Questionnaire (ACQ-NOW) was administered to measure the severity of alcohol craving at baseline, after the last rTMS session, and after 1 month of the last rTMS session	Significant reduction in the post-rTMS ACQ-NOW total score in active rTMS compared to the sham stimulation. Effect size was moderate (η^2=0.401)
Höppner et al. 2011	RCT	N=19 detoxified female patients	Patients were randomized to receive either high-frequency rTMS (20 Hz) over the left DLPFC (n = 10) or sham stimulations (n = 9) for 10 days	Obsessive-Compulsive Drinking Scale, Hamilton Depression Rating Scale, and Beck's Depression Inventory	There were no significant differences between groups with regard to alcohol craving or mood.
Herremans et al. 2012	Prospective, single-blind, sham-controlled study	N=36 hospitalized patients with alcohol dependence syndrome	After successful detoxification, patients were allocated to receive one active or one sham HF-rTMS session	Obsessive–compulsive drinking scale (OCDS)	One single-blind sham-controlled HF-rTMS session applied to the right DLPFC did not result in changes in craving (neither immediately after the stimulation session nor in patients' natural environment during the weekend)
Herremans et al. 2013	Randomized, single-blind, sham-controlled, crossover study	N=50 detoxified alcohol-dependent patients	A single right DLPFC HF-rTMS session (20 Hz, 40 trains of 1.9 seconds duration with 12 seconds interval, at 110% rMT)	Patients completed a Go-NoGo task (50% Go/50% No Go condition), Obsessive–Compulsive Drinking Scale (OCDS)	After both stimulation conditions, a significant decrease in errors commission was observed, without differences between active and sham stimulation. No significant difference was observed between active and sham stimulation on mean RT. However, only active stimulation resulted in a significant decrease in IIRTV. No effects of stimulation were found for the craving measurements.

Herremans et al. 2015	Randomized, single-blind, sham-controlled, neuroimaging study	N=26 recently detoxified alcohol-dependent patients	Initial one sham-controlled HF-rTMS session (20 Hz, 40 trains of 1.9 s duration, separated by an intertrain interval of 12 s (1560 pulses per session) at 110% rMT. This was followed by an open-label accelerated rTMS protocol such that from Wednesday until Friday, all patients received 14 active stimulations: with a between-session delay of 15 minutes: 4 on Wednesday, 5 on Thursday, and 5 on Friday, making it 15 HF-rTMS sessions in total (23,400 pulses).	fMRI alcohol cue-reactivity paradigm	General craving significantly decreased after 15 active HF-rTMS sessions. However, cue-induced alcohol craving was not altered. Brain imaging results did not show that the cue exposure affected the underlying craving neurocircuit after both one and fifteen active HF-rTMS sessions. Yet, brain activation changes after one and 15 HF-rTMS sessions, respectively, were observed in regions associated with the extended reward system and the default mode network, but only during the presentation of the event-related paradigm.
Mishra et al. 2015	Prospective, single-blind, parallel-group, active-comparator study	N=20	Patients were randomized to receive either right or left DLPFC HF stimulation. 10 sessions at 10 Hz, 20 trains, 4.9 seconds per train with 30 seconds intertrain interval)	Alcohol Craving Questionnaire (ACQ-NOW)	No main effect of group ($F_{1,18}$= 0.0001) but significant main effect of time ($F_{1,18}$= 185.91, p<0.0001, η^2=0.912). The interaction effect between group and time was not significant. There was significant reduction in craving scores in patients receiving either right or left rTMS with large effect size.
Kearney-Ramos et al. 2018	Single-blinded, within-subject, sham-controlled crossover neuroimaging study	N=24	110% resting motor threshold, 6 sessions of cTBS for each visit, 3600 pulses total over ventromedial PFC (VMPFC)	Functional MRI alcohol cue reactivity task	Cue-related functional connectivity was significantly attenuated following active cTBS versus sham cTBS (t_{23}=-5.91, FDR p<0.00001). There was no significant interaction with region of interest.
McCalley et al. 2022	Double-blind, sham-controlled neuroimaging study	N=50	10 sessions of continuous theta burst stimulation (TBS) over mPFC (left frontal pole; 1 session/day; 110% rMT, 3600 pulse/session, cue-provocation prior and during session)	Feasibility, retention, brain reactivity to alcohol cues	. Individuals that received active TBS were 2.71 times more likely to remain enrolled in the study after 3 months and 3.09 times more likely to remain sober 3 months after treatment initiation. Active TBS also led to a significantly greater reduction in brain reactivity to alcohol cues 1 and 2 months after TBS treatment.
**Deep TMS H1 Coil**					

Ceccanti et al. 2015	Double-blind RCT / monotherapy	N=18	10 sessions (5/week) of 30 trains at 20 Hz, 30 seconds intertrain interval, 120% rMT, to the mPFC	Cortisolemia and prolactinemia were evaluated as effectiveness markers. Alcohol intake and craving were considered secondary outcomes	Deep TMS significantly reduced blood cortisol levels and decreased prolactinemia, suggesting dopamine increase. Craving VAS decreased, as well as mean number of alcoholic drinks/day and drinks on days of maximum alcohol intake (DMAI).
Girardi et al. 2015	Open label / add on	N=20 abstinent patients with DSM-IV-TR AUD comorbid with previously developed dysthymic disorder	Ten patients received standard drug treatment (SDT) for AUD with add-on bilateral Deep TMS over the DLPFC (20 Hz, 120% rMT), while another 10 received SDT alone	Obsessive-Compulsive Drinking Scale (OCDS), Hamilton Depression Rating Scale (HDRS), Clinical Global Impressions scale (CGI), Global Assessment of Functioning (GAF)	At the end of the 20-session Deep TMS period (or an equivalent period in the SDT group), craving scores and depressive symptoms in the Deep TMS-Add On group dropped significantly more than in the SDT group ($p<0.001$ and $p<0.02$, respectively).
Rapinesi et al. 2015	Open label / add on	N=12 MDD patients and N=11 patients with concomitant MDD and AUD	20 Deep TMS sessions over DLPFC, 18 Hz, 120% rMT	Clinical status was assessed through the Hamilton Depression Rating Scale (HDRS) Clinical Global Impressions severity scale (CGIs), Obsessive Compulsive Drinking Scale (OCDS) in MDD+AUD, Global Assessment of Functioning (GAF)	Percent drops in HDRS and CGI scores at the end of the sessions were respectively 62.6% and 78.2% for MDD+AUD, and 55.2% and 67.1% for MDD ($p<0.001$). HDRS, CGIs, and GAF scores remained significantly improved after the 6-month follow-up. HDRS scores dropped significantly earlier in MDD+AUD than in MDD
Addolorato et al. 2017	RCT / monotherapy	N=11	12 Deep TMS sessions (3/week) over DLPFC, 20 trains per session at an intensity of 100% rMT, 50 pulses per train at a frequency of 10 Hz, and intertrain interval of 15 seconds	Clinical and single photon emission computed tomography (SPECT) evaluations of alcohol intake and dopamine transporter availability in the striatum of AUD patients were carried out after 4 weeks of Deep TMS sessions	Patients receiving the active stimulation revealed a reduction in DAT availability at the end of 4 weeks of treatment, whereas the sham-treated group did not. In addition, patients receiving the active stimulation had a decrease in alcohol intake.
**Deep TMS H8 Coil**					
Perini et al. 2019	RCT	N=56	3 weeks of 15 daily sessions of HF (10 Hz) Deep TMS or sham over the lateral prefrontal and insular cortices. Each session was preceded by craving induction (3 minutes of holding	Craving and self-reported as well as biomarker-based drinking measures were collected at baseline, during	No differences between groups in drinking patterns. There were differences in resting-state connectivity between active and sham groups at

			and smelling, but not consuming, the alcoholic beverage of choice for each participant)	treatment, and through 12 weeks. Resting-state magnetic resonance imaging (rsMRI) data and task-based MRI was used to probe brain correlates of reward processing, affective responses, and alcohol following completion of treatment.	completion of treatment, potentially indicating an ability of treatment to modify insula function.
**Deep TMS H7 Coil**					
Harel et al. 2021	Double-blind RCT / monotherapy	N=51 recently abstinent treatment-seeking patients with AUD (moderate to severe)	15 sessions over 3 weeks, followed by five sessions over 3 months of follow-up. Each session delivered 100 trains of 30 pulses at 10 Hz to the mPFC and ACC. Each session was preceded by craving induction as described by Perini et al. above	The primary predefined outcome was reduction in percentage of heavy drinking days, obtained using timeline follow-back interviews. Secondary analyses included self-reports of craving, ethyl glucuronide in urine, and brain imaging measures	Both craving and percentage of heavy drinking days during follow-up were significantly lower in the active versus sham control group (percentage of heavy drinking days = 2.9 ± 0.8% vs. 10.6 ± 1.9%, $p=0.037$). Active Deep TMS was associated with decreased resting-state functional connectivity of the dorsal ACC with the caudate nucleus and decreased connectivity of the mPFC to the subgenual ACC.

Table S3. Summary table of TMS studies in cocaine use disorder.

Study	Design	Sample	Protocol / Stimulation Parameters	Assessments	Results
**Figure-8 coil**					
Camprodon JA et al. 2007	Randomized cross-over study	N=6	Single session of high frequency (10 Hz) rTMS over left or right DLPFC	Before, immediately after, and 4 hours after rTMS, cocaine craving was measured using Visual Analogue Scale (VAS)	Right, but not left, DLPFC stimulation significantly reduced craving over time ($F_{2,10}$=11.07; p=0.0029). The reduction was 19% (13.4–24.6%) from baseline and disappeared after 4 h. The interaction of time by site of stimulation for craving was also significant ($F_{2,25}$=6.13; p=0.0068).
Politi E et al. 2008	Open label / monotherapy	N=36, all participants underwent detoxification therapy prior to the rTMS treatment	10 daily sessions of high frequency (15 Hz) rTMS over the left DLPFC (20 trains of 2 seconds with 30 seconds intertrain interval at 100% rMT)	On each stimulation day, participants underwent clinical evaluation of psychopathologic symptoms connected with craving. Every element was classified within a range of 0 (absence of symptom) to 3 (strong presence of symptom)	Repeated measures ANOVA showed that cocaine craving gradually reduced during rTMS sessions with remarkable change at the 7th session ($F_{30,270}$=4.96; p<0.0004).
Terraneo A et al. 2016	Open label RCT with rTMS vs. standard treatment (pharmacology)	N=32	Patients were randomly assigned to either the experimental group (rTMS on the left DLPFC), or to a control group (pharmacological agents) during a 29-day study (Stage 1). This was followed by a 63-day follow-up (Stage 2), during	Primary outcome was the use of cocaine during Stage 1, assessed using a urine drug screen for cocaine in the rTMS *vs.* control groups. Secondary outcomes included cocaine use during Stage 2, including comparisons to Stage 1 (within-subject	Amongst the patients who completed Stage 1, 16 were in the rTMS group (100%) and 13 in the control group (81%). During Stage 1, there were a significantly higher number of cocaine-free urine drug tests in the rTMS group

			which all participants were offered rTMS treatment.	comparisons), and cocaine craving.	compared to control (p=0.004). Craving for cocaine was also significantly lower in the rTMS group compared to the controls (p=0.038). Out of 13 patients who completed Stage 1 in the control group, 10 patients received rTMS treatment during Stage 2 and showed significant improvement with favorable outcomes becoming comparable to those of the rTMS group.
**Deep TMS H1 Coil**					
Bolloni et al. 2016	RCT / Monotherapy	N=10	12 Deep TMS sessions over 4 weeks (3/week) over bilateral PFC (10 Hz, 100% rMT)	Cocaine intake (ng/mg) was assessed by hair analysis at baseline (before treatment, T0), after 1-month (end of treatment, T1), 3 (T2), and 6 (T3) months later. All subjects received psychological support weekly.	Two-way repeated measures ANOVA did not show a significant effect of interaction between time and treatment. However, a decreasing trend in cocaine consumption in the active Deep TMS group ($F_{3,23}$=3.42; p=0.04) vs. sham ($F_{3,15}$=1.88; p=0.2) was observed in exploratory analysis with time as factor. Post hoc comparisons showed a significant reduction in the amount of cocaine detected from the onset to 3 months later (T0-T2; p=0.02) and to the end of

					treatment (T0-T3; $p=0.01$) in addicts from the active group.
Rapinesi et al. 2016	Open label / add on	N=7	12 sessions over 1 month (3/week) of high frequency (20 Hz) Deep TMS over DLPFC (20 trains at 100% rMT)	VAS was used to measure cocaine craving the week before, each week during, and one month after Deep TMS treatment	DLPFC stimulation significantly reduced craving over time ($F_{3,18}=46.154$; $p<0.001$; $\eta^2=0.88$). The reduction in craving from baseline was significant at 2 weeks ($p<0.001$) and 4 weeks ($p<0.001$) of treatment, and at week 8, four weeks after treatment interruption ($p=0.003$), although the increase in craving was significant from week 4 to 8 ($p=0.014$).
**Deep TMS H7 Coil**					

Martinez et al. 2018	RCT	N=18, all participants were actively using smoked cocaine at study entry (verified by urine toxicology) and could not be seeking treatment for their cocaine use. All subjects were admitted to an inpatient unit for the duration of the study (3 weeks)	Participants underwent 3–5 days of initial monitored abstinence, followed by self-administration sessions and high frequency (10 Hz)/low frequency (1 Hz)/sham Deep TMS over the mPFC and ACC, at 100% rMT. The procedures included: (1) baseline cocaine self-administration sessions (session 1); (2) a second cocaine self-administration session performed after 4 days (during week 1) of rTMS (session 2); (3) a third cocaine self-administration session performed at the end of 13 rTMS sessions (the end of week 3, session 3). rTMS was delivered on weekdays, over the course of 3 weeks.	Self-administration sessions, as established previously by the same group, began with a "priming" dose of smoked cocaine (12 mg). Then volunteers were presented with the choice between smoked cocaine (12 mg) and money ($5) nine times during each session. In addition, they were required to press the keyboard space bar to receive their choice. The number of presses required to obtain the dose increased using a progressive ratio schedule (e.g., 200, 400, 600, 800) to reflect a subject's motivation to obtain their choice. Outcome measure was the number of doses chosen and taken during each session. Participants were asked to rate their craving for cocaine prior to each session using VAS	The results showed a significant group by time effect (p=0.02), where the choices for cocaine decreased between sessions 2 and 3 in the high-frequency group (t=4.00, p=0.001). There was no effect of Deep TMS on cocaine self-administration in the low-frequency or sham groups. Craving was not affected by any of the condition. For all three Deep TMS conditions, the craving scores at session 3 were lower than session 2 ($F_{1,17}$=12.08; p=0.003). However, Deep TMS did not affect subsequent measures of craving $F_{2,14}$= 0.77; p=0.48).
**Deep TMS H4 Coil**					
Sanna et al. 2019	Open label RCT with iTBS vs HF rTMS	N=47	For both the HF and the iTBS protocols, subjects received 20 stimulations over 4 weeks over the bilateral PFC and insula: 10 stimulations during the 1st week, 4 stimulations during the 2nd week, 3 stimulations during	Patients were asked about the weekly amount of cocaine consumed at baseline and at the end of the treatment; cocaine consumption was evaluated twice a week by means of a commercial urine drug screen test. Craving for cocaine	Both treatments significantly reduced the intake of cocaine with no statistical differences between the two groups. Two-way ANOVA revealed a significant effect of time ($F_{1,90}$=49.97; p<0.0001) but not of

			the 3rd and 4th week. For HF protocol the simulation was at 15 Hz, 40 trains of 60 pulses each (4 s) with 15 s inter-stimulus interval for a total of 2400 pulses at 100% rMT. iTBS protocol consisted of bursts containing 3 pulses at 50 Hz repeated at 200-ms intervals for 2 s (i.e., at 5 Hz) at 80% RMT. A 2-second train of iTBS was repeated every 10 s for 190 s and 600 pulses	was assessed once a week using the cocaine craving questionnaire (CCQ-brief)	treatment ($F_{1,90}$= 0.67) or time × treatment interaction ($F_{1,90}$= 0.66). The urine test analysis revealed a significant decrease in positive tests at the end of the treatment with no difference between protocols. At the end of the treatment, 80% and 82% of the patients who received HF and iTBS protocol, respectively, had negative urine tests. Both treatments significantly reduced craving for cocaine. Two-way ANOVA revealed a significant effect of time ($F_{1,90}$ = 127.3; $p<0.0001$) but not of treatment ($F_{1,90}$ = 1.48) or time × treatment interaction ($F_{1,90}$ = 0.03). Both treatments had low numbers of dropouts and similar side-effects, safety and tolerability profiles.

Sanna et al. 2022	Retrospective analysis	N=89	Patients were treated with iTBS applied bilaterally to PFC for 20 sessions. Follow-up was performed in patients that were stably drug-free at the end of the treatment. Among these, patients who chose to undergo maintenance sessions of iTBS received one treatment a week for 1 month followed by one treatment every 2 weeks for 2 months.	Patients and their relatives/caregivers were asked to perform urine tests at the clinic or at home once a week. Data were collected at 3, 6, and 12 months.	At the end of treatment 61 (81%) abstinent patients underwent a 12-month follow-up. Among these, 27 patients chose to follow a maintenance treatment (M), whereas 34 patients chose not to adhere to a maintenance treatment (NM). Overall, among patients reaching the 12 months follow-up endpoint, 69.7% were still abstinent and 30.3% relapsed. In NM patients the drop-out rate was significantly higher than in M-ones (58.82 vs. 29.63%, p=0.04).
